# Bodychecking Rules and Concussion in Elite Hockey

**DOI:** 10.1371/journal.pone.0069122

**Published:** 2013-07-17

**Authors:** Laura Donaldson, Mark Asbridge, Michael D. Cusimano

**Affiliations:** 1 Division of Neurosurgery and Injury Prevention Research Office, St. Michael’s Hospital, University of Toronto, Toronto, Ontario, Canada; 2 Canadian Brain Injury and Violence Research Team, St. Michael’s Hospital, University of Toronto, Toronto, Ontario, Canada; 3 Department of Community Health and Epidemiology and Department of Emergency Medicine, Centre for Clinical Research, Dalhousie University, Halifax, Nova Scotia, Canada; Public Health Agency of Barcelona, Spain

## Abstract

Athletes participating in contact sports such as ice hockey are exposed to a high risk of suffering a concussion. We determined whether recent rule changes regulating contact to the head introduced in 2010–11 and 2011–12 have been effective in reducing the incidence of concussion in the National Hockey League (NHL). A league with a longstanding ban on hits contacting the head, the Ontario Hockey League (OHL), was also studied. A retrospective study of NHL and OHL games for the 2009–10 to 2011–12 seasons was performed using official game records and team injury reports in addition to other media sources. Concussion incidence over the 3 seasons analyzed was 5.23 per 100 NHL regular season games and 5.05 per 100 OHL regular season games (IRR 1.04; 95% CI 1.01, 1.50). When injuries described as concussion-like or suspicious of concussion were included, incidences rose to 8.8 and 7.1 per 100 games respectively (IRR 1.23; 95% CI 0.81, 1.32). The number of NHL concussions or suspected concussions was lower in 2009–10 than in 2010–11 (IRR 0.61; 95% CI 0.45, 0.83), but did not increase from 2010–11 to 2011–12 (IRR 1.05; 95% CI 0.80, 1.38). 64.2% of NHL concussions were caused by bodychecking, and only 28.4% of concussions and 36.8% of suspected concussions were caused by illegal incidents. We conclude that rules regulating bodychecking to the head did not reduce the number of players suffering concussions during NHL regular season play and that further changes or stricter enforcement of existing rules may be required to minimize the risk of players suffering these injuries.

## Introduction

In Canada, there were 5,629 hospitalizations due to winter sports and recreation activities in 2010–11, including 415 head injuries [1]. Mandatory helmet use in professional sport decreases incidence of head and neck injuries [2], [3]. However, protective equipment may not specifically protect against concussion [4], [5]. Recent proposals to reduce concussions in sport have recommended regulating game play in order to reduce the number of direct impacts to the head, a more difficult change to introduce [6]–[8]. In ice hockey, players use a strategy known as bodychecking to slow or stop an opposing player. Bodychecking is a frequent cause of head injury and concussion in hockey and concussion rates are higher in youth leagues that allow bodychecking [2], [9]–[12]. A ban on bodychecking has been proposed in youth hockey [9], however, aggressive play is seen as a selling point in professional leagues [13], [14].

In the National Hockey League (NHL), recent bodychecking incidents causing devastating injuries to a number of high profile players have drawn increased media attention to the importance of concussion prevention. Sponsors have called upon the NHL to address injury risks [15] and insurance companies are concerned about continuing to compensate teams for salary costs lost due to concussed players [16]. The NHL has opted to introduce rules regulating bodychecking to the head in an attempt to improve player safety. Prior to the 2010–11 season, bodychecking another player with the head as the primary point of contact was legal. In the 2010–11 season, the NHL introduced Rule 48 [17], which made targeting an opponent’s head from the blind side illegal. This rule was modified for the 2011–12 season to encompass all hits to the head, with a degree of discretion allowed on the part of the referees in determining whether the contacted player put himself at risk [18].

This study investigated concussions occurring in the 2009–10, 2010–11 and 2011–12 regular seasons in order to evaluate the effect of recent NHL rule changes regulating bodychecking. We also analyzed data from the Ontario Hockey League (OHL) to determine the concussion incidence in an elite league where any hit to the head of another player, intentional or not, has been illegal since the 2006–7 season [19]. We used injury reports from teams to determine the number of concussions, similar to a previous study on NHL players up to the 2007–2008 season [20]. Unspecified upper body injuries or injuries described as “concussion-like” were investigated using other sports media sources and were recorded as suspected concussions if the injury occurred as a result of trauma to the head and symptoms were consistent with a diagnosis of concussion [21]. The rates of violent actions and the enforcement of head checking rules in each league were determined by analyzing penalties called during game play. We hypothesized that: 1) The introduction of Rule 48, if enforced, would result in a decrease in the incidence of NHL concussions, and, 2) Concussion incidence would be higher in the NHL than in the OHL, at least before the introduction of the broad form of Rule 48 in 2011–12.

## Methods

### Study Sample and Design

A retrospective cross-sectional analysis of NHL and OHL games played during the 2009–10, 2010–11, and 2011–12 seasons was completed. This analysis involved 2211 NHL and OHL players who participated in at least one regular season game in any of the three seasons. The NHL is a professional North American hockey league consisting of 30 Canadian and American teams playing an 82 game regular season. Players must be at least 18 years old to participate. The OHL is a major junior hockey league composed of 20 Canadian and American teams playing a 68 game regular season. No Research Ethics Board approval was required for this study as all data was collected from public sources [22].

A before and after study was carried out to evaluate the impact of the introduction of Rule 48 on concussion incidence. One season prior to the introduction of Rule 48 and the two subsequent seasons where examined; in the first season Rule 48 was enforced in a narrow manner, and the second season after it was applied more broadly. OHL data, where a similar policy against checking to the head has been present since 2006, was employed as a control.

### Data Collection

#### Penalties

Penalty calls during ten weeks randomly selected from the overlapping full weeks of the OHL and NHL regular seasons were analyzed. The same weeks were analyzed for both leagues, with an average week consisted of approximately 40 NHL and 25 OHL games. All available official gamesheets for the selected dates were obtained from www.ontariohockeyleague.com or www.nhl.com and the penalty calls were recorded. Penalties were classified as: match, game misconduct, misconduct, fighting, check to the head, other aggressive, restraining, unsportsmanlike conduct, and other (e.g. bench minors, delay of game and other non-aggressive penalties).

#### Player statistics

Player statistics were obtained from The Sports Network, and official league websites with fight and salary information (www.hockeyfights.com and www.capgeek.com, respectively).

#### Concussions

For each player, the number of concussions suffered during the regular season was recorded, including suspected concussions. Suspected concussions were those described as concussion-like symptoms by the team or as concussions by multiple sports media sources (other than the team itself) and were injuries occurring as a result of trauma to the head. For descriptions of injuries, a variety of publically available sources were accessed including www.rotoworld.com, The Sports Network (www.tsn.com), sports.yahoo.com, CBSSports.com, local newspapers and official team websites. In a study of injury in professional basketball players, internet sources always provided a body part that had been injured and also provided a type of injury (e.g. sprain, laceration) 82.4% of the time [23]. No direct comparison to official injury reports was available, however, comparison with a 17-year study based on official medical reports [24] revealed similar injury patterns. For example, both studies identified a concussion rate of 0.2 per 1000 athlete exposures, and comparable rates for other injuries such as ankle injuries, the most common area injured (2.9 [23] and 3.4 [24] per 1000 athlete exposures). Facial fractures excluding the nasal bone were included in the category of suspected concussion as such injuries are caused by the application of substantial force to the head and are associated with a number of intracranial injuries [25], [26].

#### Concussion mechanisms

Concussions/suspected concussions occurring during ten week samples from the three NHL regular seasons (the same weeks used for penalty analysis) were analyzed. For NHL concussions or suspected concussions, game film publicly available from the league (NHL Vault™) was reviewed by two independent scorers. The cause of concussion was categorized as blindsiding (checking from the player’s blind side with primary contact to the head), other checking to the head, checking to the body, fighting, non-contact or collision with a teammate, hit by a stick or hit by a puck. Any secondary contact of the head or body with the boards or ice was recorded. Disagreements were resolved by a third viewer. Official NHL game summaries provided detailed information on players drawing penalties. The date of the first game missed used as the standard for the date of injury.

### Statistical Analysis

All statistical analysis was performed with STATA v12 using p<0.05 as the threshold of significance.

#### Penalties

Incidence rates and incidence rate ratios (IRRs) were calculated based on a per game basis for each league. Multinomial logistic regression was performed to determine the relative risk ratio (RRR) for each penalty class using “Other”, non-aggressive penalties as the base outcome in the NHL versus the OHL.

#### Concussions

Incidence rates per 100 games and IRRs were calculated for each league. One- and two-way analysis of variance (ANOVA) with post-hoc comparisons using Bonferroni corrections were used to determine changes over the three seasons analyzed. Medians were calculated for the number of games missed due to injury, as this data was not normally distributed. Chi-squared or Fisher’s exact tests were used to test trends in the mechanisms causing concussion over the three seasons.

#### Predictors of concussion

To determine risk factors for concussion, stepwise logistic regression was used. Risk factors included: season (2009–10, 2010–11 or 2011–12), league (NHL or OHL), age, height, weight, position (goalie, center, wing or defense), games of league experience, years of league experience, fights per 10 games, scoring (points per game), penalty minutes per game, games on the roster (games played plus games missed due to injury or suspension), whether the player had incurred a fine or suspension during the year, and the ranking of the player’s team in terms of total penalty minutes during the season (averaged for players participating for more than one team within a single season). For a subgroup analysis of NHL skaters, average on-ice minutes per game, hits per game, shots blocked per game and salary were also included. Risk factors were added to a logistic regression model using stepwise selection with a probability for inclusion of 0.20.

## Results

### Concussion Incidence in the NHL Increased from 2009–10 to 2010–11

The total number of concussions and suspected concussions (including facial fractures) suffered by players during regular season play was recorded for both leagues in all three seasons (Table 1).

**Table 1:** Concussions in the OHL and NHL.

2009–10	OHL	Concussion	23
2009–10	OHL	Suspected Concussion	6
2009–10	OHL	Facial Fracture	3
2009–10	NHL	Concussion	44
2009–10	NHL	Suspected Concussion	24
2009–10	NHL	Facial Fracture	9
2010–11	OHL	Concussion	38
2010–11	OHL	Suspected Concussion	7
2010–11	OHL	Facial Fracture	2
2010–11	NHL	Concussion	65
2010–11	NHL	Suspected Concussion	42
2010–11	NHL	Facial Fracture	13
2011–12	OHL	Concussion	42
2011–12	OHL	Suspected Concussion	19
2011–12	OHL	Facial Fracture	5
2011–12	NHL	Concussion	84
2011–12	NHL	Suspected Concussion	36
2011–12	NHL	Facial Fracture	6

Total numbers of concussions, suspected concussions and suspected concussions associated with facial fractures occurring during all of regular season play are shown for each season in the OHL and NHL.

The number of concussions versus suspected concussions did not change over the three seasons studied (1-way ANOVA: F_2, 467_ = 0.87, p>0.05). The number of games missed was higher for concussions than for suspected concussions (median of 8 for concussion, 3 for suspected concussion; 2-way ANOVA: F_2, 467_ = 3.99, p<0.05) but there was no effect of season on games missed (2-way ANOVA: F_2, 467_ = 0.10, p>0.05).

Relative to the 2010–11 season, the concussion incidence rate in the NHL was lower in 2009–10, but there was no significant difference between 2010–11 and 2011–12 (Table 2). This finding was maintained whether or not the analysis included suspected concussions. OHL concussion incidence rates were not significantly different over the seasons studied, other than an increase in concussions and suspected concussions in 2011–12 relative to 2010–11.

**Table 2:** Concussion incidence in the OHL and NHL by season.

		Concussions			+ Suspected		
	Season	Incidence/100 games	IRR	95% CI	Incidence/100 games	IRR	95% CI
NHL	2009–10	3.58	0.64*	0.42, 0.96	6.26	0.61*	0.45, 0.83
NHL	2010–11	5.28	1	N/A	9.76	1	N/A
NHL	2011–12	6.83	1.35	0.96, 1.89	10.24	1.05	0.80, 1.38
OHL	2009–10	3.38	0.60	0.35, 1.05	4.71	0.65	0.40, 1.07
OHL	2010–11	5.59	1	N/A	6.91	1	N/A
OHL	2011–12	6.18	1.18	0.74, 1.89	9.71	1.54*	1.02, 2.31

Incidence rates were calculated per 100 regular season games. IRRs for concussions and concussions plus suspected concussions were calculated relative to the 2010–11 season. Concussion incidence rate in the NHL was lower in 2009–10 than in 2010–11 (p = 0.002 for concussion and suspected concussion, p = 0.029 for concussion), but there was no significant difference between 2010–11 and 2011–12 (p = 0.727 for concussion and suspected concussion, p = 0.086 for concussion). OHL concussion incidence rates were not different between 2009–10 and 2010–11 (p = 0.074 for concussion and suspected concussion, p = 0.09 for concussion) but concussions and suspected concussion increased from 2010–11 to 2011–12 (p = 0.039, p = 0.483 for concussion only).

* indicates p<0.05 within each league relative to 2010–11.

### Concussion Incidence was Similar between the NHL and the OHL

The total concussion incidence over the 3 seasons analyzed was higher in the NHL: 5.23 per 100 NHL regular season games versus 5.05 per 100 OHL regular season games (IRR 1.04; 95% CI 1.01, 1.50). When both concussion and suspected concussion were included, these numbers rose to 8.8 and 7.1 per 100 games respectively and the difference was no longer significant (IRR 1.23; 95% CI 0.81, 1.32). Within single seasons, the incidence rates for concussions in the NHL compared to the OHL were not significantly greater with the exception of the 2010–11 season if both concussions and suspected concussions were included (Table 2), indicating that the higher overall concussion incidence in the NHL was due to the spike in suspected NHL concussions in the 2010–11 season.

### Penalties for Aggressive Actions, Including Fighting, were More Frequent in the OHL

One factor potentially affecting concussion incidence is aggressive actions, including fighting and illegal bodychecking maneuvers such as boarding and elbowing. We capture this through the number of penalties rather than the number of events. The average number of total penalties called in a game was lower in the NHL (9.4 versus 13.9 per game, IRR = 0.68; 95% CI 0.66, 0.69). The RRR for misconduct, unsportsmanlike conduct and all aggressive penalties compared to non-aggressive penalties was lower in the NHL versus the OHL, whereas restraining penalties showed the opposite pattern (Table 3). The Rule 48 check to the head penalty was enforced once in 475 NHL games analyzed for the 2010–11 season and 13 times in 465 games from 2011–12 season.

**Table 3:** Penalties by type in the OHL and NHL.

	OHL	NHL	RRR (overall)	RRR 2009–10	RRR 2010–11	RRR 2011–12
Match	0.20	0.02	0.07*	0	0.08*	0.11*
Game Misconduct	1.09	0.63	0.53*	0.66	0.66	0.34*
Misconduct	3.91	1.94	0.46*	0.38*	0.48*	0.51*
Fighting	13.9	11.2	0.74*	0.85	0.75*	0.64*
Check to the Head	1.96	0.32	0.05*	0	0.01*	0.10*
Other Aggressive	37.4	33.9	0.84*	1.01	0.76*	0.77*
Restraining	31.9	42.7	1.23*	1.38*	1.15	1.15
Unsportsmanlike	2.50	1.70	0.62*	0.66*	0.48*	0.77
Nonaggressive/Other	7.20	7.83	BASE	BASE	BASE	BASE

The proportion of the total penalty calls is shown in the first two data columns. RRRs obtained from multinomial logistic regression are relative to non-aggressive penalties for the NHL versus the OHL. Penalties from all available gamesheets were analyzed from the 10 weeks randomly selected for each season.

* indicates p<0.05.

### Most NHL Concussions Resulted from Legal Actions

The mechanism causing concussion was determined for 123 concussions or suspected concussions that occurred during a random sample of games during 10 week periods from each of the three NHL seasons. Illegal incidents, where the aggressor was assessed a penalty, fine or suspension, accounted for 28.4% of cases for concussion and 36.8% of cases for suspected concussions. In one case (0.81%), the player suffering a concussion was the player assessed a penalty. The most common penalty called was fighting (32.3%). Check to the head was the call 11.8% of the time, while other aggressive penalties accounted for 44.1% and non-aggressive penalties 11.8%. Six incidents causing concussions or suspected concussions were not assessed a penalty during game play, but were later assessed either a suspension or fine. Fewer data were available for OHL concussions, however, when a penalized incident was identified, the most common penalty type was fighting and check to the head (4 of 11 penalized incidents each, 36.4%).

**Figure 1:**
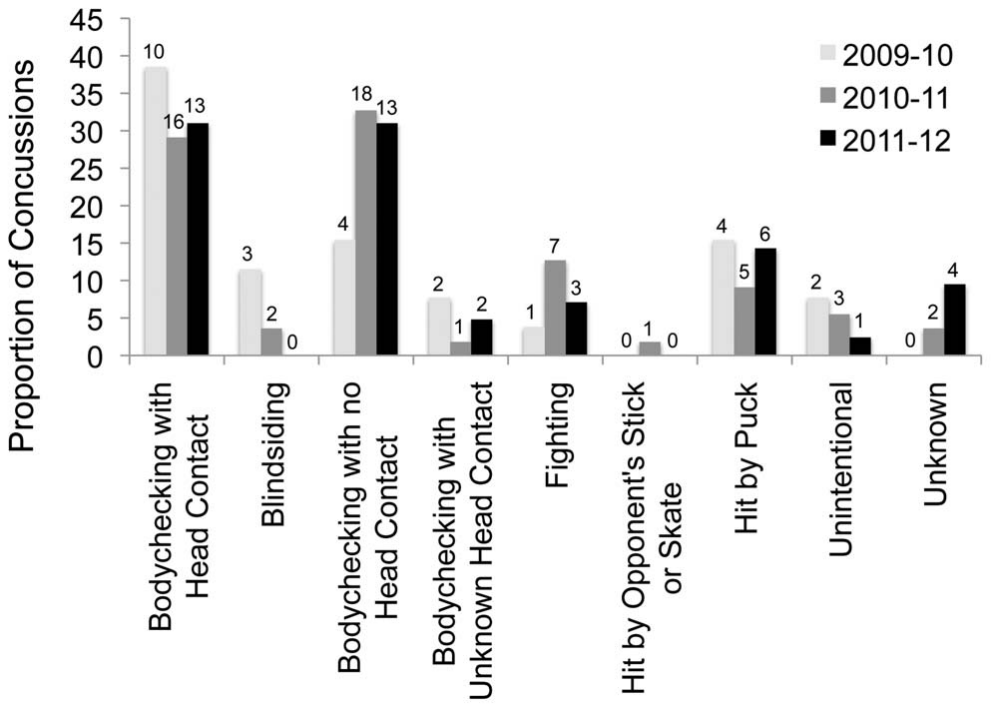
Mechanisms causing NHL concussion. The causes of NHL concussion or suspected concussion were documented for the subset of injuries occurring during the 10 randomly selected weeks for all 3 seasons. The proportion of injuries within each season caused by each mechanism is shown, with the number of injuries above each bar. Unintentional actions included tripping and colliding with a teammate. The rates of each mechanism remained constant over the seasons tested (p>0.05 for all).

The most common cause of NHL concussion was bodychecking, with and without head contact (64.2%, Figure 1). Few were caused specifically by blindsiding (4.1%). Unintentional actions such as a collision with a teammate were responsible for 4.9% of cases and an additional 12.2% were caused when players were hit by pucks. The number caused by each of these mechanisms did not change over the three seasons (χ^2^ test or Fisher’s exact tests, all p>0.05), however, the number of blindsiding injuries decreased to zero in 2011–12. 51.2% of all incidents involved a secondary contact of the head after the initial impact, most commonly to the boards or ice. Being hit in the face by a puck was the most common cause of suspected concussion associated with facial fracture (7 of 12 cases). Most of the players injured in this manner were not wearing a visor at the time (6 of 7 cases).

### Risk Factors for Concussion

When compared to wingers, goalies were less likely to suffer a concussion (crude OR = 0.47; 95% CI 0.26–0.87 for concussion; crude OR = 0.42; 95% CI 0.25–0.69 for a concussion or a suspected concussion) and defensemen were more likely to suffer a concussion (crude OR = 1.36; 95% CI 1.02–1.80 for concussion; crude OR = 1.18∶95% CI 0.93–1.48 for a concussion or a suspected concussion). The proportions of players at each position suffering concussions did not change over the three seasons analyzed (1-way ANOVA: F_2, 467_ = 0.41, p>0.05). In multiple logistic regression modeling for the outcome of any concussion or suspected concussion during the regular season, playing in the 2011–12 or 2010–11 season and more games on the team roster were associated with a higher risk (Table 4). Defenseman were also at a higher risk, but players with a greater weight were at a decreased risk. In a subgroup analysis of NHL skaters, season, more games on the roster and playing defense were again found to be associated with a higher risk of concussion. More experienced NHL skaters and those engaging in more fights were also at a higher risk of a concussion or suspected concussion, while taller and older skaters were at a lower risk. Skaters with more minutes of ice time per game were also at a lower risk.

**Table 4:** Risk factors for suffering a concussion or suspected concussion during the regular season.

Risk Factor	Adjusted OR	p value	95% CI
All Players			
2010–11 Season	1.62	0.0	1.25, 2.11
2011–12 Season	1.89	0.0	1.47, 2.44
Games on Roster	1.03	0.0	1.03, 1.04
Defenseman	1.26	0.159	0.93, 1.55
Weight	0.997	0.036	0.986, 0.999
NHL Skaters Only			
2010–11 Season	1.66	0.002	1.21, 2.29
2011–12 Season	1.70	0.001	1.25, 2.33
Games on Roster	1.03	0.0	1.03, 1.04
Defenseman	1.62	0.008	1.14, 2.31
Fights per 10 Games	1.17	0.046	1.00, 1.37
Years of Experience	1.06	0.103	0.99, 1.15
Minutes per Game	0.96	0.024	0.93, 1.00
Age	0.93	0.046	0.87, 1.00
Height	0.91	0.07	0.85, 0.98

Stepwise logistic regression for the outcome of a player suffering a concussion or suspected concussion during one full regular season was performed. Risk factors tested for all OHL and NHL players were: season (2009–10, 2010–11 or 2011–12), league (NHL or OHL), age, height, weight, position (goalie, center, wing or defense), games of league experience, years of league experience, fights per 10 games, scoring (points per game), penalty minutes per game, games on the roster (games played plus games missed due to injury or suspension), whether the player had incurred a fine or suspension during the year, and the ranking of the player’s team in terms of total penalty minutes during the season. For a subgroup analysis of NHL skaters, average on-ice minutes per game, hits per game, shots blocked per game and salary were also included.

## Discussion

NHL concussions did not decrease after the introduction of Rule 48 in 2010–11, which may be unsurprising given that blindsiding was found to be an uncommon cause of NHL concussions. The observation that concussion incidence was unchanged between 2010–11 and 2011–12, when the broader version of Rule 48 was introduced suggests that penalizing intentional checking to the head may not be effective on its own as a strategy to reduce concussion incidence. The similarity between OHL and NHL concussion rates also supports this contention, however, the unexpectedly high numbers of concussions in the OHL could be related to the higher number of fights and illegal player-to-player contact that occurred on a per game basis in the OHL. The stable rate of concussion in the OHL does suggest that the degree of awareness of concussions in these elite leagues has likely remained stable and that the lack of a reduction in concussions in the NHL is not due to increasing diagnosis. The NHL may simply need more time. A rule can have no effect unless it is enforced, and a lack of enforcement was suggested by the observation that checking to the head penalties were called 10-fold less often per game in the NHL in 2011–12 (though the OHL rule is slightly different [19], covering checking to the head “in any manner”). NHL players may also be adapting their playing and checking style only gradually to the new rules.

OHL and NHL defenseman were found to be at a higher risk of suffering a concussion than other players, perhaps due to turning their back to retrieve pucks along the boards, which leaves them vulnerable. In the NHL, taller skaters were less likely to sustain this injury, likely because shorter players are more likely to be hit in the head by another player leading with their shoulder. It is notable that fighting and bodychecking causing secondary contact of the head with the boards or ice caused more NHL concussions than blindsiding, and these incidents are not covered by head checking rules in either league. Additional changes may need to be implemented in both leagues in order to better protect the players. These may include a broader version of head checking rules to include secondary or unintentional head contact, a ban on fighting or a harsher penalty for those involved in fights, altering equipment including head and body protection, changes to ice size and rink environment, or even changes to the structure of the game to slow its pace. Although some progress has been made in enforcing penalties for rule infractions, more consistent application of infractions and the delivery of penalties with larger impacts to the player, and the team, appear warranted before one can say that the rule has been ineffectual. If third parties like insurance companies and sponsors also reinforced league direction with their actions, it is possible that more might be achieved.

The major limitation of our study is that team injury reports and publically available data were used; we did not have access to medical records and the final player diagnosis. Injuries were often clearly reported by the team as a concussion, however, we included the category of suspected concussion as teams can be reluctant to disclose that a player has suffered a concussion. Reasons for this may include a fear that a player’s head may be targeted when they return to play and the potential to lose fans when a popular player is injured with an uncertain date of return, as the recovery from concussion can be long and unpredictable. The numerous sources available that report on OHL and especially NHL players provided detailed accounts of individual injuries, and this approach has previously been characterized as very accurate in a study of injury in professional basketball players [23]. The use of media reports also has been proposed as a useful method of obtaining epidemiological data on injuries such as drowning [27], [28]. It remains possible that the actual rate of concussion is actually higher than that reported; thus our results would represent a conservative estimate. We also cannot definitively say whether the large increase in NHL concussions in 2010–11 reflected a true increase in the number of concussed players or an increase in reporting of NHL concussions by the media. It does not appear as though increased attention to the problem of player concussions has changed whether teams report these injuries as concussion, as the relative proportion that is described as concussion-like symptoms or suspicious “upper body” injuries has not changed over time. The number of games missed by concussed players has also remained constant; this indicates that any improvement in the recognition of concussion symptoms and subsequent early removal from play has not lead to faster recoveries. Lastly, concussion rates in the OHL remained stable, suggesting that awareness did not change.

One factor that we did not account for in our analyses was a previous history of concussion, which may increase susceptibility to future injury and increases the length of time injured [29]. Perhaps the association that was found between a greater number of years of NHL experience (but not greater age itself) and a higher risk of concussion is due to an increased likelihood of previous concussive or subconcussive head trauma in these players.

### Conclusion

Concussions remain a serious risk for NHL and OHL players. Despite recent actions taken by the NHL to introduce Rule 48 regulating bodychecking to the head, concussion incidence among NHL hockey players has not decreased. Both the scope and enforcement of this rule may need to be addressed in both leagues, or else other changes will need to be introduced to the game in order to reduce injury among elite hockey players.
